# Ethyl acetate fraction of *Amomum xanthioides* improves bile duct ligation-induced liver fibrosis of rat model via modulation of pro-fibrogenic cytokines

**DOI:** 10.1038/srep14531

**Published:** 2015-09-28

**Authors:** Hyeong-Geug Kim, Jong-Min Han, Jin-Seok Lee, Jong Suk Lee, Chang-Gue Son

**Affiliations:** 1 Liver and Immunology Research Center, Daejeon Oriental Hospital of Daejeon University, 22-5 Daehung-dong, Jung-gu, Daejeon, 301-724, South Korea; 2 GyeongGi Bio-Center, GSTEP, 864-1 Iui-dong, Yeongtong-gu, Suwon, Gyeonggi-do, South Korea

## Abstract

We investigated anti-hepatofibrotic effects of ethyl acetate fraction of *Ammomum xanthoides* (EFAX) using bile duct ligation (BDL)-induced hepatic fibrosis in a rat model. Male SD rats (6 weeks old) underwent BDL followed by 15 days of orall administration of EFAX (12.5, 25 or 50 mg/kg) or ursodeoxycholic acid (25 mg/kg). BDL caused animal death, ascites formation, alterations in serum biochemistries, and severe hepatic injury with excessive collagen deposition, whereas EFAX treatment significantly attenuated these effects. BDL markedly increased the pro-fibrogenic cytokines (TGF-β, PDGF-β, and CTGF) and the extracellular matrix indicators α-SMA, TIMP-1 and collagen type 1 in hepatic proteins and gene expression levels, which were notably normalized by EFAX treatment. EFAX also markedly normalized pro-fibrogenic signaling molecules including Smad2/3, Smad7, Akt, p44/42, and p38. We further explored EFAX mechanisms of actions using LX-2 cells (human derived hepatic stellate cell line). Pre-treatment with EFAX drastically attenuated the activation of α-SMA and Smad2/3, which are downstream molecules of TGF-β. These findings suggest that EFAX may be a potent anti-hepatofibrotic agent, and its corresponding mechanisms primarily involve the modulation of pro-fibrogenic cytokines.

Liver fibrosis is a serious progression in most chronic hepatic injuries including hepatic viral infections, alcohol abuse, autoimmune diseases and cholestatic liver diseases^1^. Whatever the causes of hepatic fibrosis, the pathology is characterized by excessive accumulation of extracellular matrix (ECM) via activation of hepatic stellate cells (HSCs)^2^. In the progress of hepatic fibrosis, HSCs are proliferated and activated by the stimulation of pro-fibrogenic cytokines including transforming growth factor-β (TGF-β), platelet derived growth factor-β (PDGF-β), or connective tissue growth factor (CTGF)^3^. HSC activation induces myofibroblast transition, leading to the excessive production of collagen type I and III in liver tissue^4^. In therapeutic strategies for the prevention or treatment of hepatic fibrosis, relieving or suppressing both HSC activation and proliferation have been critical targets^5^,^6^.

Among the various causes of hepatic fibrosis, incidences related to cholestatic liver injury have been steadily increasing worldwide^7^. The impairment of bile formation or bile flow leads to hepatocyte damage and then accelerates the progression into hepatic fibrosis and cirrhosis^8^. Bile duct ligation (BDL) as an animal model is commonly used to understand the pathogenesis of cholestatic lever fibrosis and or development of anti-hepatofibrotic therapeutics^9^. Failure of bile salt secretion leads to the retention of hydrophobic bile salts within hepatocytes, causing severe hepatocyte damage and the subsequent rapid activation of HSCs^10^.

Many efforts have been focused on understand the pathological features of hepatic fibrosis and developing antifibrotic therapeutics, but the pathology still remains unclear and therapeutics ineffectively. Among the various attempts to develop of anti-hepatofibrotic therapeutics, herbal plants have recently become popular candidates. *Ammomum xanthoides* (Amomi Fructus) is a well-known medicinal herb that has been used clinically to treat digestive system disorders for more than a thousand years in Asia. We previously explored the anti-hepatofibrotic effects of *Amomum xanthoides* in two chemical toxin-induced liver fibrosis models. The main pharmacological properties involved the modulation of pro-fibrogenic cytokines, as well as collagen deposition^11^,^12^. However, the pharmacological mechanism underlying the antifibrotic action of *Amomum xanthoides* at the molecular level is still unknown. Moreover, the pharmaceutical doses used were relatively high for both a water extract and a methanol fraction.

Herein, we investigate the pharmacological effects and mechanisms of the ethyl acetate fraction of *Amomum xanthoides* (EFAX) in a BDL rat model and in human derived HSC cell line, LX-2.

## Results

### Effects on the gross appearance of the liver, total body mass and survival rate

**Figure 1:**
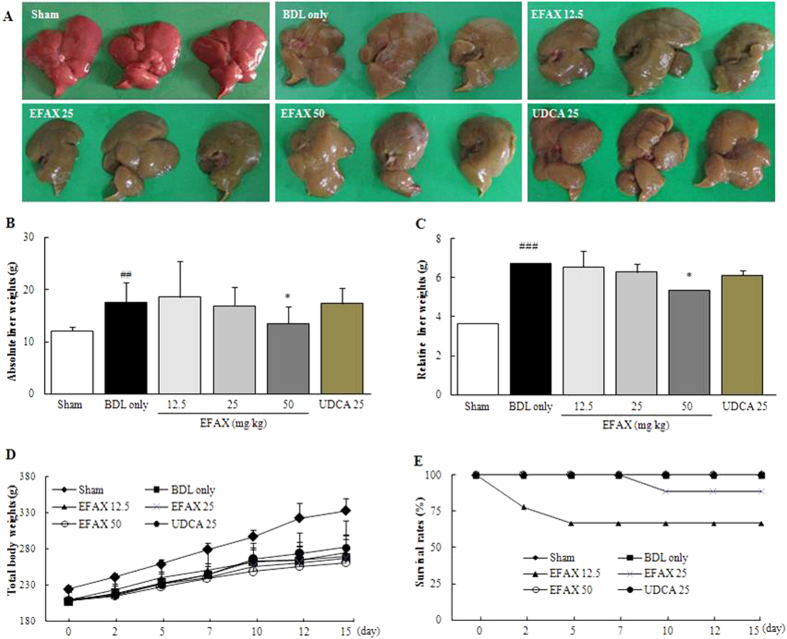
Gross finding, liver and body masses, and survival rates. Following a BDL operation, rats were orally administered distilled water, EFAX (12.5, 25 or 50 mg/kg) or UDCA (25 mg/kg) daily for fifteen days. Liver tissues examined by the naked eye (**A**), absolute liver mass (**B**), relative liver mass (**C**), total body weight changes (**D**) and survival rate (**E**). Data are expressed as the mean ± SD (*n* = 6 to 9). ^##^*p* < 0.01 and ^###^*p* < 0.001, compared with the sham group; ^*^p < 0.05 compared with the BDL only group.

The BDL only group showed a rough cirrhotic appearance in the liver surface compared with the sham group, whereas EFAX treatment (especially at 50 mg/kg) led to smooth liver surface as compared with the BDL group (Fig. 1A). The absolute and relative liver mass was considerably increased, approximately 1.5- and 1.9-fold, respectively, in BDL only group compared with the sham group. Treatment with EFAX (especially at 50 mg/kg) significantly decreased both parameters as compared with the BDL only group (*p *< 0.05, Fig. 1B,C). The total body weights of the BDL only group were lowered to approximately 20% that of the sham group, and the EFAX group didn’t recover the body weight loss (Fig. 1D). The 12.5- and 25-mg/kg EFAX groups exhibited 3 and 2 animal deaths over the course of the experiment, respectively. The other groups (BDL only, EFAX 50 mg/kg and UDCA 25 mg/kg) had no animal deaths (Fig. 1E). UDCA didn’t positively affect the total body weights or the absolute and relative liver masses.

### Effects on ascites formation and the serum levels of liver enzymes

BDL caused considerable ascites formation in the abdominal region. The BDL only group formed ascites in 7 of 9 rats, and the ascites were given a score of 1.2. The EFAX treatment notably attenuated ascites formation (5 of 6 rats in the 12.5-mg/kg EFAX group, 2 of 9 rats in the 25-mg/kg EFAX group, and 3 of 9 rats in the 50-mg/kg EFAX group). The ascites grades were also markedly reduced with scores of approximately 1.1-, 0.8-, and 0.6-score in 12.5, 25 or 50 EFAX groups, respectively (*p* < 0.05 for the 50-mg/kg EFAX group, Table 1). The serum levels of total bilirubin, aspartate transaminase (AST) alanine transaminase (ALT) gamma-glutamyl transpeptidase, alkaline phosphatase (ALP), and total cholesterol levels in the BDL only groups were higher approximately 78.0-, 4.1-, 2.5-, 49.1- 1.8-, and 1.6-fold higher than that of the sham group. The EFAX treatment (25 or 50 mg/kg) these levels (*p* < 0.05 for 50 mg/kg EFAX group in total bilirubin, for 25-and 50-mg/kg EFAX group in AST, GGT and total cholesterol; *p* < 0.01 for 50 mg/kg-EFAX group in ALT and ALP; Table 1). UDCA treatment considerably reduced the GGT, AST, and ALT serum levels.

### Effects on histopathology, collagen deposition, and lipid peroxidation in hepatic tissue

**Figure 2:**
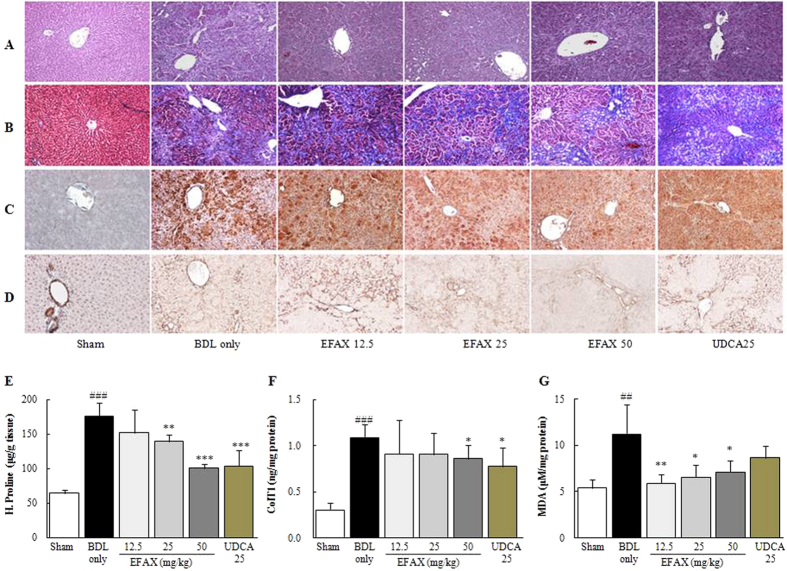
Histological examinations and hepatic tissue collagen production and lipid peroxidation. Following a BDL operation, rats were orally administered distilled water, EFAX (12.5, 25 or 50 mg/kg) or UDCA (25 mg/kg) daily for fifteen days. Representative photomicrographs of liver sections processed for hematoxylin & eosin (H&E) staining (**A**), Masson’s trichrome staining (**B**), immunohistochemistry against TGF-β1 (**C**) and α-SMA (**D**). Photographs were obtained by light microscopy at 100× magnification. Hepatic tissue levels of hydroxyproline (**E**), collagen type 1 (**F**) and MDA (**G**). Data are expressed as the mean ± SD (*n* = 6 to 9). ^##^*p* < 0.01 and ^###^*p* < 0.001, compared with the sham group; ^*^p < 0.05, ^**^p < 0.01 and ^***^p < 0.001, compared with the BDL only group.

The histopathology findings indicated severe liver injury, including bridging necrosis and wide infiltration of inflammatory cells around the central vein in the BDL only group, while EFAX markedly decreased these symptoms (Fig. 2A). The hepatic tissue collagen depositions were assessed using Masson’s trichrome staining. Collagens accumulated considerably in BDL only group (stained in blue), whereas EFAX treatment markedly inhibited accumulation (Fig. 2B). Immunohistochemistry showed that both TGF-β1 and α-smooth muscle actin (SMA) were strongly enhanced in BDL only group (stained in red-brown), while EFAX treatment notably attenuated these signals as compared with the sham group (Fig. 2C,D). In the BDL only group, the levels of hydroxyproline and collagen type 1 in the hepatic tissue levels were drastically increased 2.7- and 3.6-respectively, compared with the sham group, whereas EFAX treatment significantly decreased these levels (*p* < 0.05 for the 50-mg/kg EFAX group in collagen type 1; *p* < 0.01 for 25-mg/kg EFAX group in hydroxyproline; *p* < 0.001 for 50-mg/kg EFAX group in hydroxyproline, respectively, Fig. 2E,F). A marker of lipid peroxidation, hepatic tissues MDA content, was notably increased approximately 2.1-fold in the BDL only group as compared with the sham group, whereas EFAX treatment significantly decreased hepatic MDA levels as compared with the BDL only group (*p* < 0.05 for the 25- and 50-mg/kg EFAX groups; *p* < 0.01 for the 12.5-mg/kg EFAX group; Fig. 2G). Administration with UDCA similarly affected the histopathological examinations and the levels of hydroxyproline contents and collagen type 1, but not the hepatic tissue MDA content.

### Effects on the protein and gene expression levels of pro-hepatofibrotic cytokines in hepatic tissue

**Figure 3:**
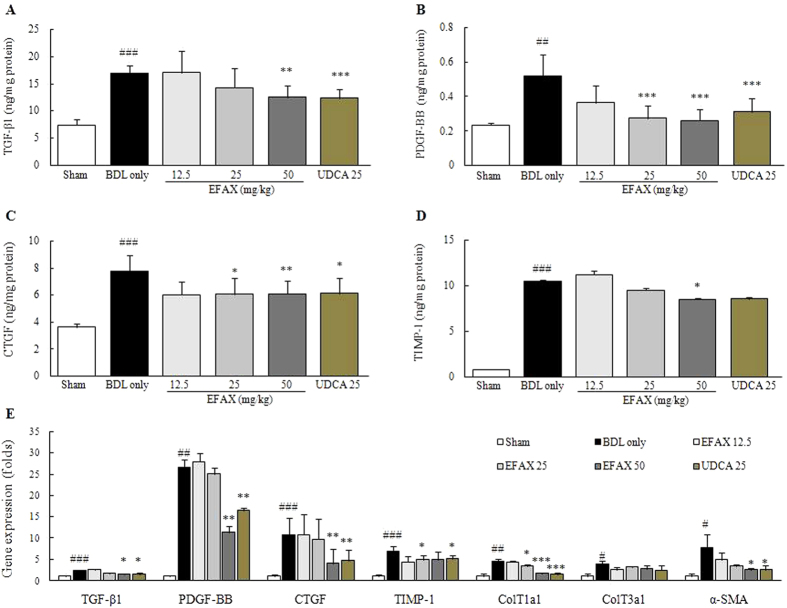
Protein and gene expression levels of pro-hepatofibrotic cytokines. Following a BDL operation, rats were orally administered distilled water, EFAX (12.5, 25 or 50 mg/kg) or UDCA (25 mg/kg) daily for fifteen days. Quantitative analysis of hepatic protein levels of TGF-β1 (**A**), PDGF-BB (**B**), CTGF (**C**) and TIMP-1 (**D**) were performed using ELISA method. Gene expression levels of TGF-β1, PDGF-BB, CTGF, TIMP-1, collagen type 1a1, collagen type 3a1, and α-SMA were performed using real-time PCR (**E**). Data are expressed as the mean ± SD (*n* = 6 to 9). ^##^*p* < 0.01 and ^###^*p* < 0.001, compared with the sham group; ^*^p < 0.05, ^**^p < 0.01 and ^***^p < 0.001, compared with the BDL only group.

The hepatic tissue levels of pro-fibrogenic cytokines including TGF-β1, PDGF-BB and CTFG were approximately 2.3-, 2.2- and 2.1-fold higher in the BDL only group than that of the sham group, respectively. EFAX treatment, however, significantly decreased the elevation of these cytokines as compared with the BDL only group (*p* < 0.05 for the 25 mg/ mg/kg-EFAX group in CTGF; *p* < 0.01 for 50 mg/kg-EFAX group in TGF-β1 and CTGF; *p* < 0.001 for 25- and 50 mf/kg-EFAX group in PDGF-BB; Fig. 3A–C). The hepatic protein level of tissue inhibitors of matrix metalloproteinase (TIMP)-1 was also markedly increased in the BDL only group approximately 13.8-fold as compared with the sham group, whereas EFAX treatment (especially at 50 mg/kg) significantly attenuated the level of TIMP-1 as compared with the BDL only group (*p* < 0.05, Fig. 3D).

The hepatic gene expression levels of the pro-fibrogenic cytokines TGF-β1, PDGF-BB, and CTGF were drastically up-regulated by 2.5-, 26.6-, and 10.7- fold, respectively, as compared with the sham group, whereas EFAX significantly normalized these expression levels as compared with the BDL only group (*p* < 0.05 for 50-mg/kg EFAX group in TGF-β1; *p* < 0.01 for 50-mg/kg EFAX group in CTGF and PDGF-BB; Fig. 3E). The gene expression levels of collagen type 1a1, collagen type 3a1, α-SMA and TIMP-1 were also up-regulated in the BDL only group approximately 4.6-, 3.9-, 7.7- and 7.0-fold, respectively, as compared with the sham group, whereas these alterationslevels were significantly normalized by administration with EFAX (*p* < 0.05 for 25 mg/kg-EFAX group in collagen type 1a1, collagen type 3a1 and TIMP-1, for 50 mg/kg-EFAX group in α-SMA; *p* < 0.01 for 12.5 mg/kg-EFAX group in collagen type 2a3; *p* < 0.001 for 50 mg/kg-EFAX group in collagen type 1a1; Fig. 3E). Administration with UDCA showed similar effects in both hepatic protein and gene expression levels.

### Effects on HSC activation signaling in hepatic tissue

**Figure 4:**
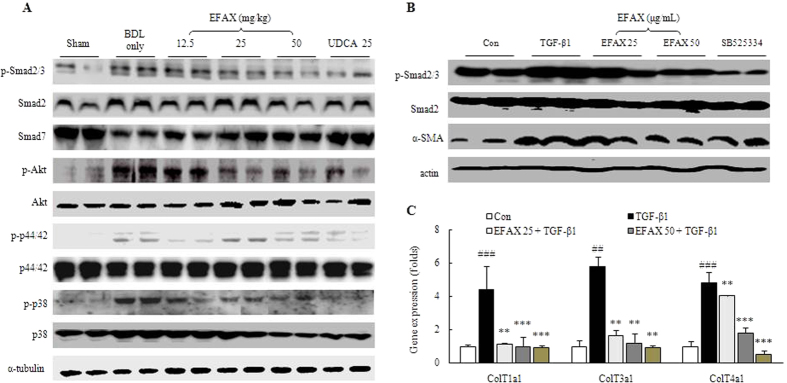
Protein and gene expression analyses for the signaling of hepatic stellate cell (HSC) activation. Following a BDL operation, rats were orally administered distilled water, EFAX (12.5, 25 or 50 mg/kg) or UDCA (25 mg/kg) daily for fifteen days. Protein expression levels of TGF-β1 signaling pathway related molecules in hepatic tissue were performed using western blot analysis (*n* = 6 to 9) (**A**). Western blot analysis of Smad 2/3 signaling and α-SMA were conducted in LX-2 cell (*n* = 4 for each group) under various concentrations of EFAX (0, 25, or 50 μg/mL), or SB 525334 (10 μM) with TGF-β1 (1 ng/mL) (**B**). Gene expression analysis of collagen type 1a1, 3a1 and 4a1 were performed using real-time PCR in LX-2 cells (*n* = 4 for each group) under the same condition (**C**). For the gene expression data, ^##^*p* <  < 0.01 and ^###^*p* < 0.001 compared with the control group; ^**^p < 0.01 and ^***^p < 0.001 compared with the TGF-β1 only group.

In the BDL only group, levels of the TGF-β1 signaling pathway-related proteins phospho-Smad2/3 and Smad7 were drastically altered as compared with the sham group, whereas administration with EFAX dramatically normalized these levels as compared with the BDL only group (Fig. 4A). EFAX also significantly normalized alterations of the phosphatidylinositol 3(PI 3) -kinase downstream signaling protein phospho-Akt, which was at abnormal status in the BDL only group. Two members of the mitogen-activated protein kinase (MAPK) subfamily, the extracellular signal-regulated kinases (ERK)-1 (also known as p44/42) and p38 were considerably activated in the BDL only group, whereas administration with either EFAX or UDCA notably normalized those both kinases (Fig. 4A).

### Effects on TGF-β signaling and α-SMA in LX-2 cells

Pre-treatment with TGF-β1 (1 ng/mL) considerably increased the protein levels of both phospho-Smad2/3 and α-SMA as compared with control group. Pre-treatment with EFAX (at 25 and 50 μg/mL), however, notably normalized these levels in TGF-β1-stimulated LX-2 cells (Fig. 4B). The mRNA expression levels of collagen type 1a1, 3a1 and 4a1 increased approximately 4.4-, 5.8- and 4.8-fold, respectively, as compared with the control group. Pre-treatment with EFAX significantly normalized these levels as compared with TGF-β1 only group (*p* < 0.01 for 25 μg/mL-EFAX group in collagen type 1a1, 3a1 and 4a1, for 50 μg/mL-EFAX group in collagen type 3a1; *p* < 0.001 for 50 μg/mL-EFAX group in collagen type 1a1 and 4a1; Fig. 4C). Pre-treatment with SB 525334, a known antagonist of Smad2/3, also inhibited mRNA expression and reduced the protein levels of collagens, phospho-Smad2/3 and α-SMA in the LX-2 cells.

### Comparative activity of EFAX and its three major components in HSC-T6 cells

**Figure 5:**
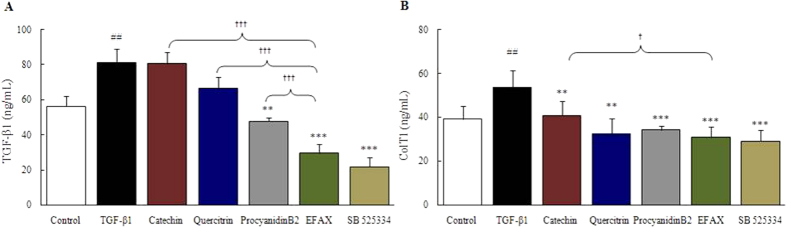
Comparative activity of EFAX and three of its components on the productions of TGF-β1 and collagen type 1. HSC-T6 cells were pre-treated (for 12 hours) with catechin, quercitrin, procyanidin B2 (each 2 μg/mL), or EFAX (10 μg/mL), and then stimulated with TGF-β1 (1 ng/mL, for 24 hours). The levels of TGF-β1 (**A**) and collagen type 1 (**B**) in the culture media were measured. Data are expressed as the mean ± SD (*n* = 4 for each group). ^#^*p* < 0.05 and ^##^*p* < 0.01 compared with the control group; ^**^p < 0.01 and ^***^p < 0.001 compared with the TGF-β1 only group; ^†^p < 0.05 and ^†††^p < 0.001 compared with the three compound groups.

Stimulation with TGF-β1 (1 ng/mL) increased both TGF-β1 and collagen type 1 (approximately 1.4-fold for each) as compared with the control group, whereas pre-treatment with procyanidin B2 (2 μg/mL) significantly decreased the only the level of TGF-β1 level (*p* < 0.01, Fig. 5A). Pre-treatment with any of the three major EFAX components (catechin, quercitrin, or procyanidin Bs) significantly decreased collagen type 1 as compared with TGF-β1 only group. (*p* < 0.01 for catechin and quercitrin, *p* < 0.001 for procyanidin B2, respectively, Fig. 5B). Comparing the anti-hepatofibrotic effects between EFAX and its major three components, EFAX showed the strongest anti-hepatofibrotic effects (*p* < 0.05 *vs.* catechin in collagen type 1, *p* < 0.01 *vs.* catechin and *p* < 0.001 *vs.* quercitrin and procyanidin B2 in TGF-β1; Fig. 5A,B). SB 525334 also showed the anti-hepatofibrotic effects on the TGF-β1 stimulated HSC-T6 cells.

## Discussion and Conclusion

Cholestatic liver injury, which is provoked by biliary obstruction, progresses hepatic fibrosis or cirrhosis clinically^13^. Although cholestatic liver disease is rare, some evidences show that its prevalence is steadily increasing, estimated at 1 to 3 in every 4,000 adult males, and 1 in every 10,000 in live births^7^,^10^. Cholestatic liver injury is initiated by bile acid retention in hepatic tissue, resulting in continuous inflammation and hepato-cellular necrosis or apoptosis^8^. This stimulus induces HSC activation, and eventually, the development of and then finally develops hepatic fibrosis^14^. No efficient therapeutics for cholestatic liver injury has been developed to date. Thus we investigated the antifibrotic effects of EFAX and its corresponding mechanisms using a BDL rat model^15^, a clinical relevant animal model for studying the molecular basis of cholestatic liver fibrosis^16^.

In our experiment, BDL drastically altered liver surface appearance, such as the loss of a smooth and shiny color. Both absolute and relative liver masses were drastically increased, but body weight markedly decreased after BDL operation. Treatment with EFAX (especially at 50 mg/kg), however, significantly attenuated these changes (Fig. 1A–D). A typical pathological feature in severe hepatic fibrosis, ascites formation considerable was in the BDL only group, whereas EFAX treatment (especially at 50 mg/kg) significantly improved this symptom (Table 1). Bile flow obstruction was completed blocked by BDL, as evidenced by the dramatic elevation of total bilirubin, GGT, ALP, and total cholesterol serum levels, while EFAX administration significantly ameliorated these levels (Table 1). These conditions directly led to severe inflammatory reactions^17^, as shown by abnormal elevations of serum AST and ALT levels, and the inflamed cell infiltration of hepatic tissue in our results. The anti-inflammatory effects of EFAX in the histopathological findings were in accordance with the significant amelioration of liver enzyme levels (Fig. 2A and Table 1).

Fifteen days of biliary obstruction led to a moderate degree of hepatic fibrosis, as demonstrated by Masson’s trichrome staining (stained in blue) and, the elevation of hydroxyproline and type 1 collagen hepatic tissue levels. In the BDL model, oxidative stress is well known to be involved in hepatocyte destruction and the liver fibrosis process^18^,^19^. As we expected, BDL considerably increased hepatic tissue MDA content (an end product of lipid peroxidation). Administration with EFAX drastically reduced hepatic tissue oxidation and collagen depositions (Fig. 2B,E–G). With respect to hepatic fibrosis, continuous hepatic damage results in fibrotic alterations in the hepatic tissues. HSCs, which are primarily responsible for liver fibrosis, play a critical role in the development of liver fibrosis. During fibrotic progression, the HSCs transform into myofibroblast-like cells and initiate ECM production via the release of fibrogenic cytokines, including TGF-β, PDGF-β, and CTGF^20^,^21^. Among the pro-fibrogenic cytokines, TGF-β can directly activate HSCs and cause the expression of PDGF-β and CTGF receptors in the liver^22^,^23^. PDGF-β is a potent mitogen and activator of HSCs, resulting in ECM production^24^,^25^. Additionally, CTGF mediates TGF-β-induced ECM synthesis^26^. In the present study, BDL markedly activated HSCs, as shown by immunohistochemical staining for the enhancement of TGF-β1 and α-SMA signals (stained in red brown), while EFAX treatment markedly inhibited these enhanced signals (Fig. 2C,D). TIMP-1 negatively affects the treatment of hepatic fibrosis by preventing ECM degradation and participating in ECM remodeling^27^. BDL also drastically increased TIMP-1 levels in hepatic tissue, while EFAX treatment significantly decreased the hepatic protein levels of TGF-β1, PDGF-BB, CTGF, and TIMP-1 (Fig. 3A,D). The effect of EFAX on pro-fibrogenic cytokines, TIMP-1, α-SMA, and a variety of collagens was in accordance with hepatic gene expression levels (Fig. 3E).

In the progression of hepatic fibrosis, TGF-β excessively contributes to the enhancement of the fibrogenic process in and *autocrine* and *paracrine* manner^28^,^29^. TGF-β expression in HSCs is initiated by phosphorylation of Smad2 and Smad3 (a known co regulator of Smad)^30^. Smad2 is activated by binding TGF-β receptor I and TGF-β receptor II to TGF-β, allowing the direct activation of HSCs^31^. Smad3, together with it co-regulator Smad, mediates ECM production when HSCs are activated^32^. In contrast, Smad7 (a known inhibitor of Smad/Smad e complexes), inhibits HSC trans-differentiation and the attenuation of fibrotic changes^33^,^34^. Thus, the regulation of Smad family is thought to be a target for the development of hepatic fibrosis therapy. Moreover, the proliferation of activated HSCs is also a critical step in the progression of liver fibrosis. HSC proliferations is initiated via the activation of PI 3-kinase and ERK signaling. These molecules are the down-stream of CTGF and TIMP-1 expression^35^,^36^,^37^.

To elucidate the molecular mechanisms of EFAX, we further explored the above molecular pathways. As expected, BDL operation drastically increased Smad2/3, but markedly decreased Smad7, whereas EFAX administration dramatically normalized these changes (Fig. 4A). Those results were also well mirrored by the Akt, p44/42, and p38 hepatic protein levels (Fig. 4A). To further investigate the antifibrotic mechanisms of EFAX, we also used TGF-β1-stimulated, human derived HSCs using LX-2 cells. Both phospho-Smad2/3 and α-SMA were considerably attenuated by EFAX pre-treatment under the TGF-β1-stimulated activation condition (Fig. 4B). The effects of EFAX on the above proteins agreed well with the gene expression of levels of collagen type 1, 3, and 4 (Fig. 4C).

*Amomum xanthoides*, also known as Ammomi Fructus, is a dried ripe fruit that belongs to the *Zingiberaceae* family^11^. Previous studies have conclusively shown that *Amomum xanthoides* has potent antioxidant and anti-inflammatory effects^38^,^39^. To date, several active compounds have been isolated from *Amomum xanthoides,* including volatile oils,, saponins, and flavonoids^11^,^40^,^41^. Chemical analysis of these compounds, however, remains deficient. Therefore, in the current study, we chemically characterized of EFAX using UHPLC/LC-MS (Fig. S2-A). A total of three different classes of active compounds, particularly flavonoid-derived chemical compounds, were detected including, catechin, procyanidin B2, and quercitrin (Fig. S2-B). Of these three chemical compounds, procyanidin B2 is most abundant components of EFAX (Fig. S2-C). According to previous studies, the procyanidin B2 and catechin exhibited potent antioxidant, anti-inflammatory, and anti-hepatofibrotic effects in various animal models^42^,^43^,^44^. To estimate the antifibrotic activity of EFAX with those active compounds, we performed further experiments using TGF-β1-stimulated HSC-T6 cells. As expected, levels of both TGF-β1 and collagen type 1 increased, and procyanidin B2 was the most potent inhibitor of these markers. Procyanidin B2 comprises 0.759% of EFAX, and a 5-fold larger dose of EFAX was significantly (1.7-fold) more effective than procyanidin B2 alone, especially for TGF-β1 (Fig. 5A,B).

These findings strongly emphasized the potential of EFAX as an anti-hepatic therapeutic agent. Furthermore, we previously reported the anti-hepatofibrotic effects of the methanol fraction and a water extract of *Amomum xanthoides* using chemical toxin-induced hepatic fibrosis models^11^,^12^. The present study, however adapted a rigorous animal model and a relatively samaller dose (25 mg/kg for EFAX) than used previously (100 mg/kg for others). Additionally, the current study also presented in detail the mechanistic actions of EFAX using various molecular methods.

In conclusion we demonstrated that EFAX modulates TGF-β-related signaling, primarily via the Smad2/3 and Smad7 signaling pathways, in BDL-induced liver fibrosis condition. Our data suggest that EFAX has antifibrotic effects against cholestatic liver injury, confirming the potential of EFAX in drug development of anti-hepatofibrotic therapeutics.

## Methods

### Materials

*Amomum xanthioides* was obtained from an herbal pharmaceutical company (Jeong-Seong Drugstore, Daejeon, Rep. of Korea). After obtaining EFAX (at a final yield of 0.19% (w/w)), its chemical compositionwas analyzed using UHPLC/LC-MS analysis (See Figs S1 and S2 online). The reagents for the present study were as follows: Histofine (Nichirei Biosciences, Tokyo, Japan); hydrochloric acid and phosphoric acid (Kanto Chemical Co., Inc., Tokyo, Japan); n-butanol (J.T. Baker, Phillipsburg, NJ); diaminobenzidine (DAB) (Abcam, Cambridge, UK); Mayer’s hematoxylin, methanol and isopropanol (Wako Pure Chemical Industries, Osaka, Japan); TRI reagent (Invitrogen, Carlsbad, CA); goat anti-human CTGF antibody, CTFG standard solution, rabbit anti-human CTGF antibody, and anti-rabbit IgG-HRP (Santa Cruz Biotechnology, Santa Cruz, CA); TGF-β1 (R&D Systems, Minneapolis, MN); SB 525334 (TOCRIS Bioscience, Bristol, UK); and catechin, quercitrin, and procyanidin B2 (Kyeong-Buk, South Korea). All other materials were purchased from Sigma-Aldrich (St. Louis, MO).

### Animals and experimental design

Fifty-one specific-pathogen-free male Sprague-Dawley rats (six-weeks old, 190–210 g) were purchased from Koatech (Gyeong-Gi, South Korea). The rats were acclimated in an environmentally controlled room at 22 ± 2 °C with a 55 ± 10% relative humidity in a 12-hour light/dark cycle. The rats were fed commercial pellets and tap water ad libitum for one week. Experiments were designed and performed strictly in accordance with the Guide for the Care and Use of Laboratory Animals and approved by the Institutional Animal Care and Use Committee of Daejeon University. After seven days of acclimation, the rats underwent BDL or a sham operation under anesthesia with ketamine (100 mg/kg, i.p., Yuhanpharmacy, Gyeong-Gi, South Korea)^45^. BDL- and sham-operated rats freely accessed a standard rat pellet diet and tap water *ad libitum* for 3 days. On the fourth day, except for the sham group (n = 6), all BDL rats were randomly divided into five groups of 9 rats each: the BDL group (no drug treatment), the EFAX groups (EFAX at 12.5, 25, or 50 mg/kg), and the UDCA group (25 mg/kg). EFAX or distilled water was given by gastric gavage daily for 15 days. Body weight was measured every three days, and the survival rates were monitored daily throughout the experiment. After 15 days, all of the rats were sacrificed under ether anesthesia after 12 hours of fasting. Whole blood was collected from the abdominal aorta, and liver tissue was removed and weighed immediately.

### Assessment of ascites index

The extents of ascites formation was graded as follows: ‘0’ for no ascites, ‘1’ for mild ascites (less than 3 mL), ‘2’ for moderate ascites (3 mL to less than 6 mL), and ‘3’ for severe ascites (over than 6 mL)^46^.

### Serum biochemistry analysis

Serum was separated by centrifugation (3,000 × g, 15 min) following the clotting of the remaining blood. Total bilirubin, ALT, AST, GGT, ALP, and cholesterol were determined using an Auto Chemistry Analyzer (Chiron, Emeryville, CA).

### Serum biochemistry analysis

Serum was separated by centrifugation (3,000 × g, 15 min) following clotting of the remaining blood. Total bilirubin, ALT, AST, GGT, ALP, and total cholesterol were determined using an Auto Chemistry Analyzer (Chiron, Emeryville, CA).

### Histopathological findings and immunohistochemical staining

A portion of liver tissue in 10% formalin solution was re-fixed in Bouin’s solution. The paraffin-embedded liver tissue was sectioned (4-μm thickness), and H&E and Masson’s trichrome staining were performed. Immunohistochemical analysis for α-SMA and TGF-β1 were performed. The liver tissue sections were de-paraffinized, hydrated and heated in a citrate buffer for antigen retrieval at 100 °C for 15 min and then treated with normal serum for 30 min. Next, the slides were treated with anti-TGF-β1 mouse mAb or anti-α-SMA mouse mAb (1:200; Abcam, Cambridge, UK) overnight. After washing three times with PBS, the tissues were incubated with the secondary antibody, *N*-Histofine Simple Stain MAX PO, and DAB. After counterstaining with Mayer’s hematoxylin, the slides were examined under an optical microscope (Leica Microsystems, Wetzlar, Germany).

### Hydroxyproline and collagen type 1 levels

The level of collagen production was determined by measuring the hydroxyproline content in liver tissue according to a previously described method^47^. Briefly, liver tissue (200 mg) was homogenized in 2 mL of 6 N HCl and incubated overnight at 110 °C. After passage of the acid hydrolysates through filter paper (Toyo Roshi Kaisha, Tokyo, Japan), 50 μL of samples or hydroxyproline standards in 6 N HCl were air-dried. The dried samples were dissolved in methanol, and then 1.2 mL of 50% isopropanol and 200 μL of chloramine-T solution were added, followed by incubation at room temperature for 10 min. Ehrlich’s solution (1.3 mL) was added, and the samples were incubated at 50 °C for 90 min. The final reaction product was read at 558 nm using a spectrophotometer (Cary 50, Vaian, Victoria, Australia).

Hepatic tissue levels of collagen type1 were evaluated using a commercial kit (C-terminal pro-peptide of type 1 collagen from the QUIDEL Corporation, San Diego, CA).

### Lipidperoxidation level

The level of lipid peroxidation in hepatic tissue was determined by measuring malondialdehyde (MDA) using the thiobarbituric acid reactive substance (TBARS) method as described previously^48^.

### Pro-fibrogenic cytokines and TIMP-1 levels

Hepatic tissue levels of other pro-fibrogenic cytokines and TIMP-1 were measured using a commercial ELISA kit (TIMP-1, TGF-β1, and PDGF-BB from R&D Systems, Minneapolis, MN). The hepatic tissue levels of CTGF were manually measured using an ELISA method^49^.

### Western blot analysis

The expression levels of HSC activation-related proteins in hepatic tissue and LX-2 cells were evaluated by western blot.

Liver tissue (approximately 200 mg) was homogenized in radioimmunoprecipitation assay (RIPA) buffer. A total of 40 μg of each protein was separated by 10% polyacrylamide gel electrophoresis and transferred to polyvinylidene fluoride (PVDF) membranes. After blocking in 5% skim milk, the membranes were probed overnight at 4 °C with primary antibodies (Samd2/3, phospho-Samd2/3, Samd7, Akt, phospho-Akt, p44/42, phospho-p44/42, p38, phospho-p38, and α-tubulin).

For western blots in LX-2 cells, cells were cultured in 100-mm petri dishes with 5% FBS in RPMI medium. Cells were pre-incubated with EFAX (0, 25, or 50 μg/mL) or SB 525334 (10 μM) for 12 hours, and then TGF-β1 (1 ng/mL) was added to the same culture dishes. The cells were collected after 30 min (for Samd2/3, phospho-Samd2/3, Samd7 and phospho-Samd7) or 24 hours (for α-SMA) for further incubation, and protein was extracted using the RIPA buffer. A total of 40 μg of each protein was separated by 10% polyacrylamide gel electrophoresis and transferred to PVDF membranes. After blocking in 5% skim milk or 5% BSA, the membranes were probed overnight at 4 °C with primary antibodies (Samd2/3, phospho-Samd2/3, Samd7, α-SMA, and β-actin).

The membranes were washed and incubated for 2 h with HRP-conjugated anti-rabbit antibody. Western blots were visualized using an enhanced chemiluminescence (ECL) kit.

### Quantitative real-time PCR analysis

Total RNA in liver tissue or LX-2 cells was extracted using QIAzol reagent (Qiagen, Valencia, CA). cDNA was then synthesized from total RNA (2 μg) in a 20-μL reaction using a High-Capacity cDNA Reverse Transcription kit (Ambion, Austin, TX).

For mRNA expression in LX-2 cells, the cells (5 × 10^6^ cells) were seeded in a 100-mm petri dish in 10 mL RPMI with 5% FBS and incubated overnight at 37 °C and 5% CO_2_. The cell culture medium was then changed to serum-free DMEM. The cells were pre-treated with EFAX (25, 50, or 100 μg/mL) or SB 525334 (10 μM) for 12 hours, and then TGF-β1 (1 ng/mL) was added. After 6 hours, total RNA was extracted as described above.

Real-time PCR was performed using SYBRGreen PCR Master Mix (Applied Biosystems, Foster City, CA, USA), and PCR amplification was performed using a standard protocol with the IQ5 PCR Thermal Cycler (Bio-Rad, Hercules, CA, USA). For data analysis, the gene expression levels were compared with those of β-actin as a reference gene.

### Cellular levels of TGF-β1 and collagen type 1

HSC-T6 cells were cultured in 24-well cell culture plates with 10% FBS in DMEM medium. The cells were pre-incubated with EFAX (10 μg/mL), catechin, procyanidin B2, quercitrin (each to 2 μg/mL) or SB 525334 (10 μM) for 12 hours, and then TGF-β1 (1 ng/mL) was added to the same culture dishes. After 24 hours of TGF-β1 treatment, the cell culture medium was harvested for measuring TGF-β1 and type 1 collagen levels.

### Statistical analysis

Results are expressed as the means ± standard deviations (SD). The statistical significance of differences between groups was analyzed by one-way analysis of variance (ANOVA), followed by a Student’s unpaired t-test. In all analyses, values of *p* < 0.05, *p* < 0.01 or *p* < 0.001 were considered statistically significant.

## Additional Information

**How to cite this article**: Kim, H.-G. *et al.* Ethyl acetate fraction of *Amomum xanthioides* improves bile duct ligation-induced liver fibrosis of rat model via modulation of pro-fibrogenic cytokines. *Sci. Rep.* **5**, 14531; doi: 10.1038/srep14531 (2015).

## Supplementary Material

Supplementary Information

## Figures and Tables

**Table 1:** Formation of abdominal ascites and serum levels of liver enzymes.

Items	Sham	BDL only	EFAX 12.5	EFAX 25	EFAX 50	UDCA 25
Ascites formation	—	7/9	5/6	2/9	3/9	7/9
Ascites grade (score)	—	1.2 ± 0.9^##^	1.2 ± 0.8	0.8 ± 1.3	0.6 ± 0.8^*^	1.0 ± 1.0
Total bilirubin (g/dL)	0.1 ± 0.0	7.8 ± 1.1^###^	8.5 ± 1.5	7.0 ± 1.2	6.6 ± 0.2^*^	6.9 ± 0.6
AST (IU/dL)	154.3 ± 38.2	628.8 ± 213.1^##^	591.0 ± 315.6	346.3 ± 113.9^*^	347.8 ± 25.9^*^	358.3 ± 41.7^*^
ALT (IU/dL)	36.2 ± 6.5	91.7 ± 16.7^###^	85.2 ± 34.3	66.8 ± 27.2	61.3 ± 6.3^**^	51.9 ± 7.5^***^
GGT (IU/dL)	2.7 ± 1.4	130.8 ± 12.3^###^	118.5 ± 49.7	100.2 ± 51.6	83.2 ± 39.0^*^	52.8 ± 9.5^***^
ALP (IU/dL)	705.7 ± 149.9	1309.3 ± 150.7.6^##^	1545.3 ± 536.7^##^	1160.7 ± 187.6	1035.2 ± 125.6^**^	36.9 ± 2.8
Total cholesterol (mg/dL)	66.2 ± 108.8	108.8 ± 17.0^###^	126.8 ± 13.9	100.0 ± 25.7	89.3 ± 10.0^*^	87.0 ± 23.9

Data are expressed as the mean ± SD (*n* = 6 to 9). ^##^*p* < 0.01 and ^###^*p* < 0.001 compared with the sham group; ^*^p < 0.05, ^**^p < 0.01 and ^***^p < 0.001 compared with the BDL-only group. AST: aspartate transaminase, ALT: alanine transaminase, GGT: gamma-glutamyl trans peptidase, and ALP: alkaline phosphatase.
