# Supplementary material for: Ethyl acetate fraction of Amomum xanthioides improves bile duct ligation-induced liver fibrosis of rat model via modulation of pro-fibrogenic cytokines
Source: Sci Rep. 2015 Sep 28;5:14531. doi: 10.1038/srep14531 (PMC4585957; doi:10.1038/srep14531)

Ethyl acetate fraction of *Amomum xanthioides* improves bile duct ligation-induced liver fibrosis of rat model via modulation of pro-fibrogenic cytokines

Hyeong-Geug Kim[1,a], Jong - Min Han[1,a], Jin-Seok Lee[1], Jong Suk Lee[2] and Chang-Gue Son[1,*]

Supplementary method

Preparation of EFAX and fingerprinting analysis. After identification of Amomum xanthioides by a professor with a herbology specialty in Oriental medical college of Daejeon University. The Amomum xanthioideswas washed using tap water at twice and rinsed with distilled water (DW), then completely dried at oven-drying for overnight (60 °C) for overnight. After then, a 1 kg of Amomum xanthioideswere cut into pieces by grinder-mixer and extracted in 10 L of absolute methanol for 7 days with shaking. At the 7th day 100 mL distilled water was added into 900 mL methanol and acquired the menthanolic extract layer. The extracts were successively fractionated with petroleum ether three times (3 × 1 L). The 100 mL of petroleum ether fraction was further mixed with distilled water 900 mL (3 × 1 L), and isolated the ethylacetate fraction. Finally, a part of 100 % ethyl acetate soluble fraction (EFAX) were dried and lyophilized used for this study. The final extraction of EFAX gave a yield of 0.19 % (w/w), and was dissolved in 2% ethanol storing at -20 °C (Fig. S1). Fingerprinting for the reproducibility of the EAFX and compositional analysis of putative compounds were conducted using ultra-high-performance liquid chromatography-tandem mass spectrometry (UHPLC-MS/MS). A 5-mg aliquot of the EFAX sample was dissolved in 1 mL of 90% methanol, and the solution was filtered (0.45 µm). The 10 µL of EFAX sample solution was subjected to UHPLC-MS using an LTQ Orbitrap XL linear ion-trap MS system (Thermo Scientific Co., San Jose, CA) equipped with an electrospray ionization source. Separation was performed on an Accela UHPLC system using an Acquity BEH C18 column (1.7 µm, 100 × 2.1 mm; Waters, Milford, MA, USA). The column was eluted at a flow rate of 0.4 mL/min using water (in 0.1% formic acid) and acetonitrile (in 0.1% formic acid), which were used as mobile phases A and B, respectively, with the following gradients: 0-1 min, 0-1% B in A; 1-7 min, 1-100% B in A; 7-10 min, 100-1% B in A (linear gradient). The compositional analysis of EFAX was conducted using a photodiode array at 200 - 600 nm. The full-scan mass spectra were acquired at 150 - 1500 m/z in positive and negative modes. An Orbitrap analyzer was used for high-resolution mass data acquisition with a mass resolving power of 30,000 FWHM at 400 m/z. Tandem mass (MS/MS) spectra were acquired in data-dependent mode by

collision-induced dissociation. The quantitative analysis of the major three compounds in EFAX including the procaynidin B2, catechin, and quercitrin were performed using UHPLC-MS (Fig. S2).

Primer sequences of mRNA for hepatic tissue and LX-2 cells. For investigating of mRNA expressions, the primers and their sequences which were we used as follows α-SMA, collagen type 1 alpha 1 (ColT1A1), collagen type 1 alpha 2 (ColT1A2), TGF-β1, PDGF-β, CTGF, tissue inhibitor of metalloproteinases-1 (TIMP-1), tissue inhibitor of metalloproteinases-2 (TIMP-2), matrix metalloproteinase-2 and 9 (MMP-2 and 9) and β-actin were as follows (forward and reverse, respectively):were as follows (forward and reverse, respectively): α-SMA, AAC ACG GCA TCA TCA CCA ACT and TTT CTC CCG GTT GGC CTT A; ColT1A1, CCC AGC GGT GGT TAT GAC TT and GCT GCG GAT GTT CTC AAT CTG; ColT1A2, CCC AGA GTG GAA GAG CGA TTA and GCT GCG GAT GTT CTC AAT CTG; TGF-β1, AGG AGA CGG AAT ACA GGG CTT T and AGC AGG AAG GGT CGG TTC AT; PDGF-β, ACC ACT CCA TCC GCT CCT TT and TGT GCT CGG GTC ATG TTCA A; CTGF, GGC ACT TTG GCT CGC ATC ATA GTT G and GTG TGT GAT GAG CCC AAG GA; TIMP-1, ATG GAG AGC CTC TGT GGA TAT GTC and AGG CAG TGA TGT GCA AAT TTC C; TIMP-2, GAG CCT AAA CCA CAG GTA CCA GAT and GTC CAT CCA CAG GCA CTC ATC; MMP-9, TCG AGG GAC GCT CCT ATT TGT and CCA TAT TTT CTG TCT GTG TCG TAG TCA; and β-actin, CTA AGG CCA ACC GTG AAA AGA T and GAC CAG AGG CAT ACA GGG ACA A, respectively. Moreover, the primers and their sequences of LX-2 cell were as follows (forward and reverse, respectively): ColT1a1, CAA CAC AGT GAT TGA ATA CAA AAC CA and ACG TCG AAG CCG AAT TCC T; ColT3a1GCC AGA ACC ATG CCA AAT ATG and GCA CAA CAT TCT CCA AAT GGA A; ColT4a1; TGC ATC ACG AAA TGA CTA CTC GTA and ACA CAC AGC ACA CCT ACT AAT AAA TGG; and β-actin, GGC ACC ACA CCT TCT ACA AT and GCC TGG ATA GCA ACG TAC AT.

Supplementary figure legends

Figure S1. Flow chart of ethylacetate fraction of *Amomum xanthoides* (EFAX).

Figure S2. Chemical constitutions and quantitative analysis of ethyl acetate fraction of *Amomum xanthoides* (EFAX) using ultra-high-performance liquid chromatography-tandem mass spectrometry (UHPLC-MS/MS) chromatogram. EFAX and three reference standards were subjected to UHPLC analysis (A), and its three main compounds were observed in the high-resolution mass spectra; proacyanidin B2 (m/z 579.17, $[M+H]^+$), catechin (m/z 291.09$[M+H]^+$) and quercitrin (m/z 303.05$[M+H]^+$) (B). The quantitative analysis of EFAX were performed (C).

Figure S3. Full-length gel images for western blotting results. Full-length gel images for Fig. 4-A and Fig. 4-C.

Supplementary figures

Fig. S1

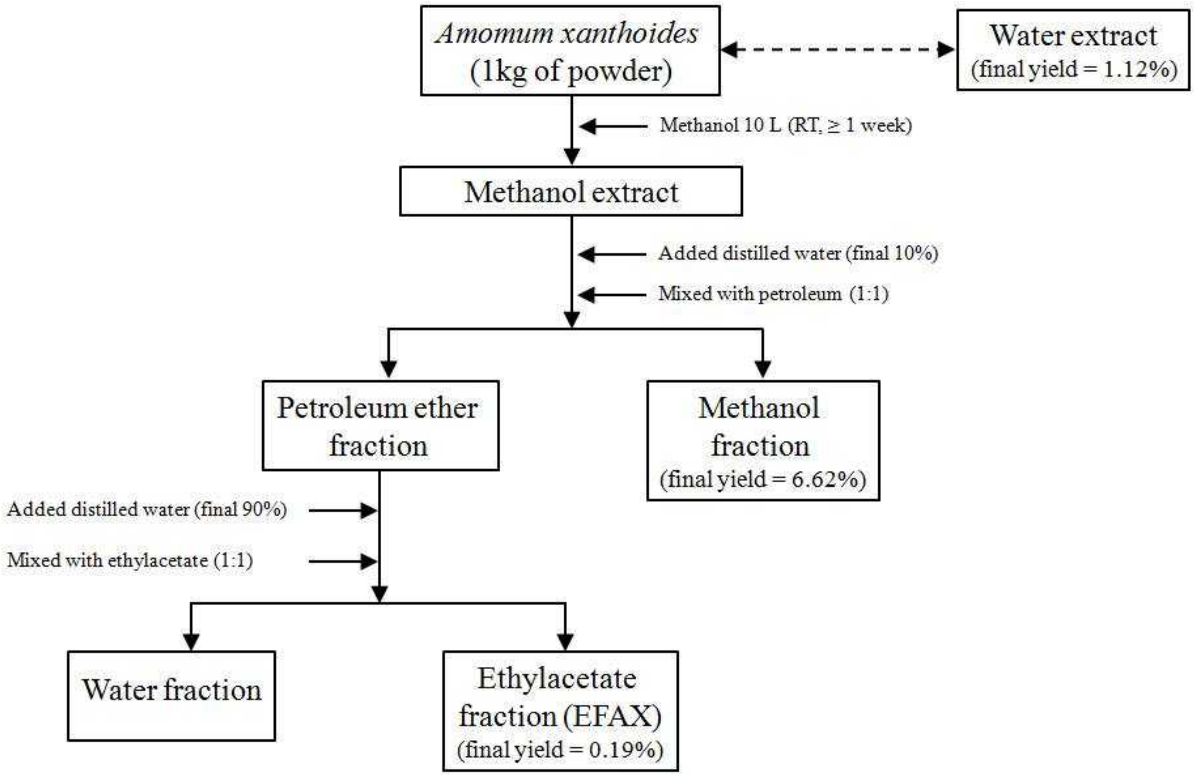

Fig. S2

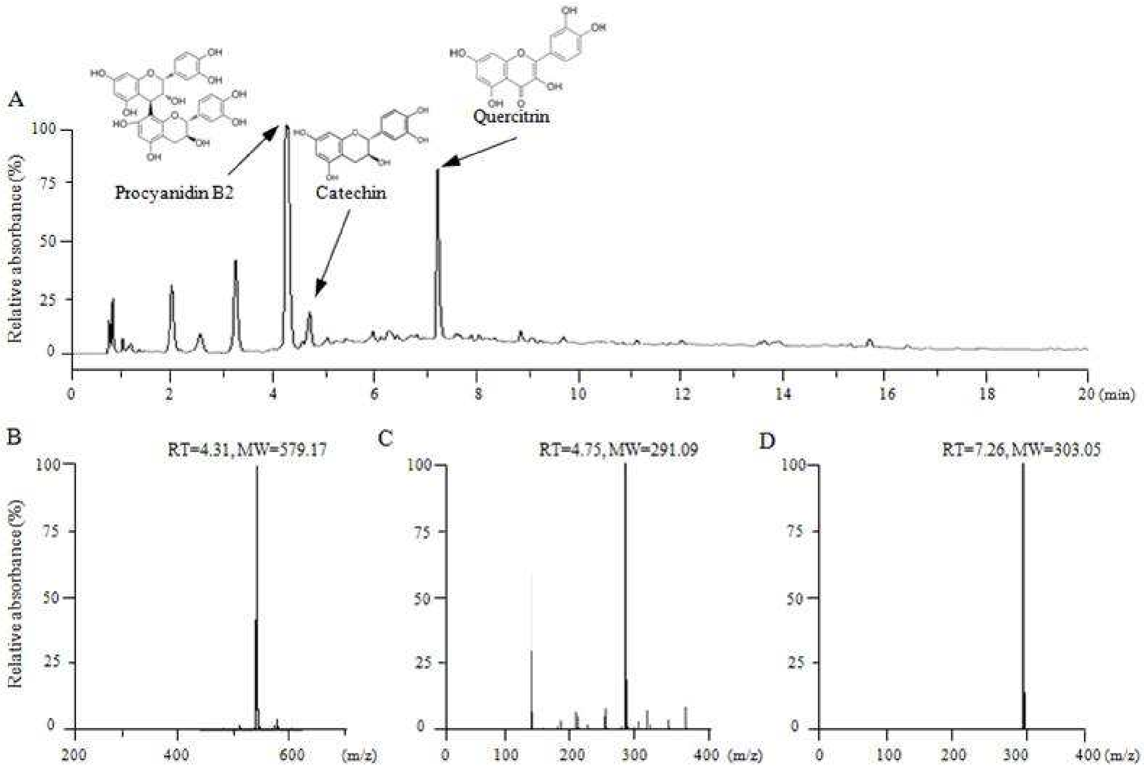

C

Compounds	Retention time (min)	Mean ± SD (μg/mg)
Procyanidin B2	4.31	7.59 ± 0.06
Catechin	4.75	0.91± 0.05
Quercitrin	7.23	5.97 ± 0.09

Fig. S3

Fig.4A

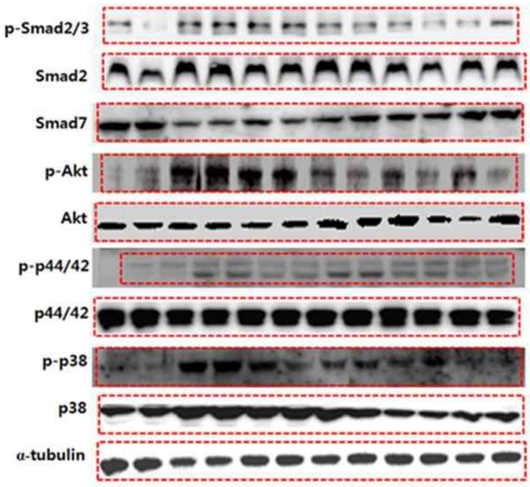

Fig.4C

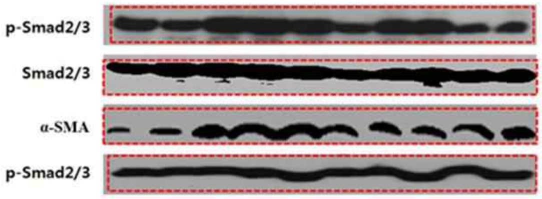

Supplement: Supplementary Information [file srep14531-s1.pdf]
